# Linear ubiquitin chains: enzymes, mechanisms and biology

**DOI:** 10.1098/rsob.170026

**Published:** 2017-04-26

**Authors:** Katrin Rittinger, Fumiyo Ikeda

**Affiliations:** 1 Molecular Structure of Cell Signalling Laboratory, The Francis Crick Institute, 1 Midland Road, London NW1 1AT, UK; 2 Institute of Molecular Biotechnology (IMBA), Dr Bohr-gasse 3, 1030 Vienna, Austria

**Keywords:** ubiquitin, RBR E3 ligases, LUBAC, OTULIN, immune responses, inflammatory signalling

## Abstract

Ubiquitination is a versatile post-translational modification that regulates a multitude of cellular processes. Its versatility is based on the ability of ubiquitin to form multiple types of polyubiquitin chains, which are recognized by specific ubiquitin receptors to induce the required cellular response. Linear ubiquitin chains are linked through Met 1 and have been established as important players of inflammatory signalling and apoptotic cell death. These chains are generated by a ubiquitin E3 ligase complex called the linear ubiquitin chain assembly complex (LUBAC) that is thus far the only E3 ligase capable of forming linear ubiquitin chains. The complex consists of three subunits, HOIP, HOIL-1L and SHARPIN, each of which have specific roles in the observed biological functions of LUBAC. Furthermore, LUBAC has been found to be associated with OTULIN and CYLD, deubiquitinases that disassemble linear chains and counterbalance the E3 ligase activity of LUBAC. Gene mutations in HOIP, HOIL-1L and OTULIN are found in human patients who suffer from autoimmune diseases, and HOIL-1L mutations are also found in myopathy patients. In this paper, we discuss the mechanisms of linear ubiquitin chain generation and disassembly by their respective enzymes and review our current understanding of their biological functions and association with human diseases.

## 1. Introduction

Post-translational modifications of proteins extend their functional landscape and allow rapid changes in their behaviour in response to stimuli without the need for protein synthesis de novo. Ubiquitination is one of the most diverse forms of post-translational modification. It plays crucial roles in nearly every type of cellular function including protein degradation, DNA damage responses, trafficking and intracellular signalling. Its diversity springs from its ability to modify target proteins either with a single or multiple single ubiquitin molecules (mono- or multi-monoubiquitination) or with polyubiquitin chains, in which individual ubiquitin molecules are linked to one another via their C-terminal carboxyl group and either a lysine side chain or the N-terminal amino group of methionine 1 (Met 1). In total, eight different types of homotypic polyubiquitin chains are formed plus many different combinations of mixed and branched ubiquitin chains [1]. The last type of homotypic polyubiquitin chains identified were the linear, also called Met 1-linked, chains in which a peptide bond connects the ubiquitin molecules within the oligomer. In 2006, a multi-subunit complex termed linear ubiquitin chain assembly complex (LUBAC) was identified to be responsible for their synthesis [2] and it was later shown that they are required for the activation of the nuclear factor kappa-light-chain-enhancer of activated B cells (NF-κB) transcription factor and play important roles in immune and inflammatory signalling processes [3,4]. Since then, their functional roles have been extended to include regulation of cell death, T- and B-cell development, mouse embryonic development, heat tolerance in flies, and cancer and autoimmune diseases in humans [5–10].

**Figure 1.**
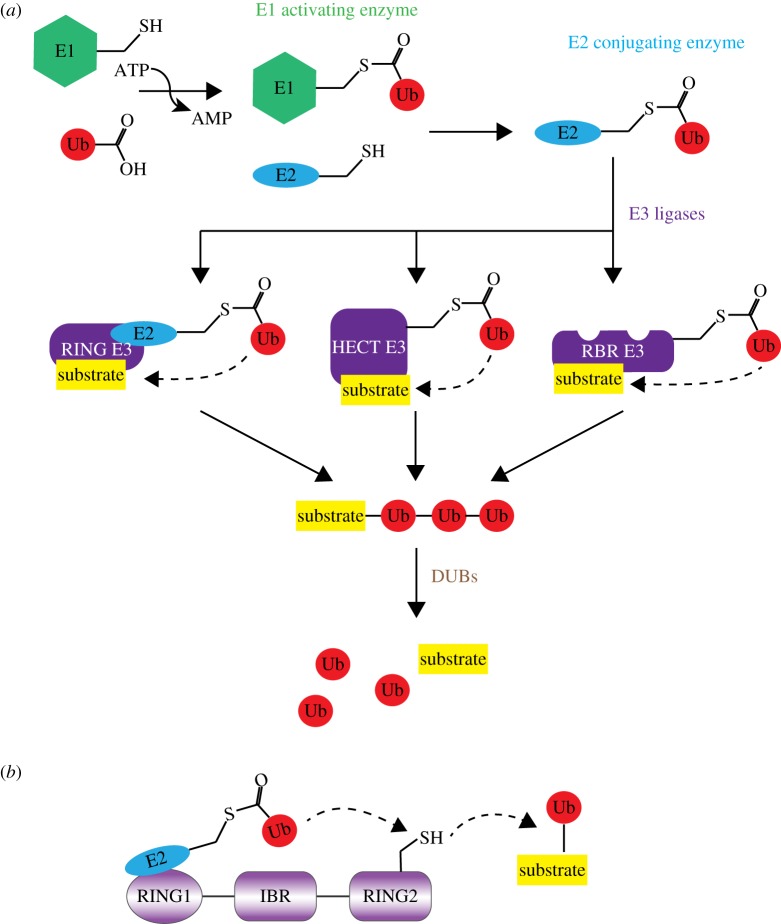
The ubiquitination cascade. (*a*) Schematic depiction of the ubiquitin conjugation system showing the ATP-dependent activation of ubiquitin and thioester formation with E1 and its subsequent transfer onto E2. Transfer onto the substrate is catalysed by E3 ubiquitin ligases, which are divided into three classes: RING, HECT and RBR ligases. Ubiquitination is reversible and ubiquitin chains are removed from substrates by DUBs. (*b*) Domain organization of the RBR catalytic module showing the RING1, IBR and RING2 domains. The E2∼Ub conjugate is recognized by the RING1 domain and transferred to a conserved cysteine in RING2 to form a thioester intermediate before the final transfer of ubiquitin onto the substrate.

Modification of proteins with ubiquitin requires the activity of three different enzymes that act in a sequential cascade that includes E1 ubiquitin-activating enzymes, E2 ubiquitin-conjugating enzymes and E3 ubiquitin ligases (figure 1*a*) [11]. E3 ligases are the key determinants of the ubiquitination process and confer specificity as they select the target protein and in some cases also the type of ubiquitin modification [12–14]. This process is reversible and ubiquitin can be removed from a target by deubiquitinating enzymes (DUBs) [15,16]. Often polyubiquitin chain synthesis and cleavage occur in unison, thereby ensuring that the correct type of ubiquitin modification is available at the required time and place.

E3 ubiquitin ligases catalyse the transfer of ubiquitin onto the substrate using different mechanisms and, based on this property, have been classified into three different subfamilies (figure 1*a*). Really interesting new gene (RING)-type ligases play a more indirect role and bring the substrate and ubiquitin-loaded E2 (E2∼Ub) together, and ubiquitin transfer occurs directly from the E2 onto the substrate [12–14]. By contrast, the homologous to the E6AP carboxyl terminus (HECT)-type ligases take the ubiquitin from the E2 to form a thioester intermediate before its final transfer onto the substrate [13,14,17]. E3 ligases of this family determine the topology of the polyubiquitin chain formed, independent of the cognate E2 [17]. A third class of E3 ligases combines properties of both these types and employs a RING domain (RING1) to initially recognize the ubiquitin-loaded E2, which is subsequently transferred to a conserved active-site cysteine in a RING2 domain to form a thioester intermediate, before the final, E2-independent ubiquitin transfer onto the substrate. This mechanism is adopted by the RING-between-RING (RBR) family of E3 ligases (figure 1*b*) [18–20]. Unlike other types of homotypic ubiquitin chains that can be synthesized by multiple HECT E3s and E2-RING E3 combinations, linear ubiquitin chains are only produced by the multi-subunit ligase LUBAC, which is a member of the RBR family of E3s.

The last few years have seen a big increase in our understanding of mechanistic and structural features of LUBAC activity and the physiological role of linear ubiquitin chains in different organisms. In this review, we will focus on these recent advances and discuss our current understanding of the determinants and regulators of linear chain synthesis by LUBAC and cleavage by DUBs and their role in the regulation of a multitude of cellular processes.

## 2. Composition of LUBAC

**Figure 2.**
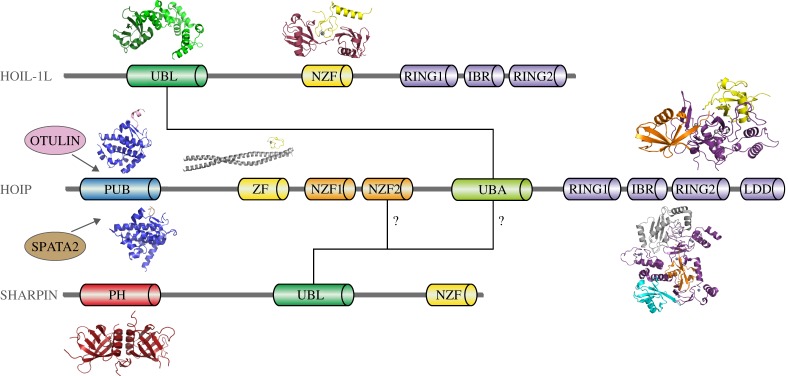
Composition of LUBAC. Schematic of the domain composition of HOIP, HOIL-1L and SHARPIN. The domains mediating the interaction between subunits are highlighted. The UBL domain of HOIL-1L binds the UBA domain of HOIP, while the interaction between SHARPIN and HOIP is less well defined and may include the NZF2 or UBA domains of HOIP, as indicated by question marks. Structural information exists for a number of subdomains of LUBAC components and their complexes with binding partners. Structures are shown where available and include the HOIL-1L UBL/HOIP UBA complex (4DBG), the HOIL-1L NZF/linear diUb complex (3B08), HOIP PUB/OTULIN PIM (4OYK, 4P0W), HOIP PUB/SPATA2 PIM (5LJN), HOIP ZF/NEMO UBAN (4OWF), HOIP RING2-LDD/ubiquitin (4LJO), HOIP RBR/UBE2D2-ubiquitin (5EDV) and the PH domain of SHARPIN (4EMO).

When LUBAC was discovered in 2006, it was initially thought to be composed of only two subunits termed HOIL1-interacting protein (HOIP)/RNF31 and haem-oxidized IRP2 ubiquitin ligase1L (HOIL-1L)/RBCK1, which associate into a high-molecular-weight complex of unknown stoichiometry [2]. Since then, work by multiple groups has revealed that the composition of LUBAC is more elaborate and contains an additional non-catalytic subunit called Shank-associated RH domain-interacting protein (SHARPIN) [21–23]. Moreover, the HOIP subunit of LUBAC has been shown to associate with regulatory proteins including the linear chain-specific OTU domain DUB with linear linkage specificity (OTULIN)/Gumby/FAM105B and the adaptor protein spermatogenesis-associated protein 2 (SPATA2), which links LUBAC to another DUB, CYLD, which cleaves linear and Lys 63-linked polyubiquitin chains [24–27]. This multi-protein machinery is held together by defined protein–protein interactions as illustrated in figure 2, some of which have been characterized structurally [27–30]. Nevertheless, it is currently unknown if any of the individual constituents of LUBAC are present in multiple copies and if the stoichiometry of the complex may be important for its physiological function. Furthermore, HOIP, HOIL-1L and SHARPIN contain ubiquitin-binding domains (UBDs) of varying specificity [4,21,31]. Nevertheless, how binding to different types of polyubiquitin chains contributes to LUBAC stability, localization and/or regulation of catalytic activity is still unknown.

Interaction between the HOIP and HOIL-1L subunits is mediated by their respective ubiquitin-associated (UBA) and ubiquitin-like (UBL) domains, which form a non-canonical UBA–UBL complex as revealed in their crystal structure (figure 2) [28]. The UBL domain of HOIL-1L adopts the typical ubiquitin fold, whereas the UBA of HOIP forms a nine-helix bundle that includes a three-helix bundle consisting of α6, α7 and α8 that is similar to canonical UBA domains. Intriguingly, in the crystal HOIP UBA makes contacts with three different molecules of HOIL-1L UBL that all recognize different binding surfaces on HOIP. Only one of these involves the Ile 44 hydrophobic patch that is normally involved in UBL-mediated interactions. However, binding studies combined with deletion mapping showed that complex formation occurs with a 1 : 1 stoichiometry in solution and involves helices α6, α7, α8 and α9, which recognize a surface on the UBL that is opposite the canonical UBA-binding surface. SHARPIN also employs its UBL domain to bind HOIP. However, the region of HOIP mediating this interaction is unclear and both UBA and NZF2 have been suggested to be involved [21–23].

The N-terminal portion of HOIP contains a PUB domain, which mediates the interaction with the PUB interaction motifs (PIMs) of OTULIN and SPATA2 (figure 2). Crystal structures of the HOIP PUB domain bound to the PIMs of OTULIN and SPATA2 have revealed that they both recognize the same binding site in HOIP and hence that their interaction is mutually exclusive [27,29,30]. PIMs contain a conserved tyrosine and its phosphorylation has been shown to abrogate the interaction with OTULIN *in vitro*, suggesting a mechanism for how the interaction might be regulated. However, a physiological relevance of phosphorylation has not been shown so far nor has a kinase capable of phosphorylating OTULIN been identified. The recent discovery of SPATA2 as a binding partner of HOIP solved the conundrum that CYLD was known to play a functional role in LUBAC signalling and required the PIM-binding capability of the PUB domain of HOIP [32,33] but that no direct interaction between the two proteins could be detected *in vitro*. Instead, it has now been shown that SPATA2 itself contains a PUB domain that unlike other PUBs does not recognize PIMs but instead interacts with CYLD to connect this DUB to LUBAC [26,27]. The biological role of these interactions between LUBAC components and DUBs are described in detail in §6.

## 3. The linear ubiquitin chain synthesis machinery

The HOIP subunit of LUBAC contains all the catalytic machinery required to synthesize linear ubiquitin chains with high specificity [2,3]. This activity is located in its C-terminal portion within the RBR domain plus a C-terminal extension referred to as the linear ubiquitin chain-determining domain (LDD) that is specific to HOIP [34,35]. RBR domains have a conserved domain structure that consists of an N-terminal RING1 domain that recognizes the ubiquitin-loaded E2, a central in-between RING (IBR) domain of yet unknown function and a C-terminal RING2 domain that harbours the catalytic cysteine, which forms the thioester intermediate during ubiquitin transfer (figure 1*b*) [18]. Recent structural and biochemical studies have allowed first insight into individual steps of the ubiquitin transfer reaction from E2 to E3 (HOIP) and onto a ubiquitin substrate, and have provided a mechanistic explanation for the *in vitro* and *in vivo* observed chain linkage specificity [34–37].

To modify a target with a polyubiquitin chain, monoubiquitination must occur first to initiate a chain, which subsequently can be extended. Originally, it was suggested that HOIP, possibly in conjunction with another LUBAC subunit, directly recognizes the target and attaches the first ubiquitin for chain initiation after which ubiquitin becomes the substrate during chain extension [3,38,39]. More recently, however, an alternative mechanism was proposed, in which the protein target, such as the IκB kinase (IKK) subunit NEMO, is modified by a Lys 63-linked chain first (by an as yet unidentified E3), which is then extended by LUBAC with a linear chain [40]. In such a model, the only substrate of LUBAC is ubiquitin itself. At present, it is not clear if these models are mutually exclusive or if a given stimulus determines if homotypic linear or mixed chains are produced.

**Figure 3.**
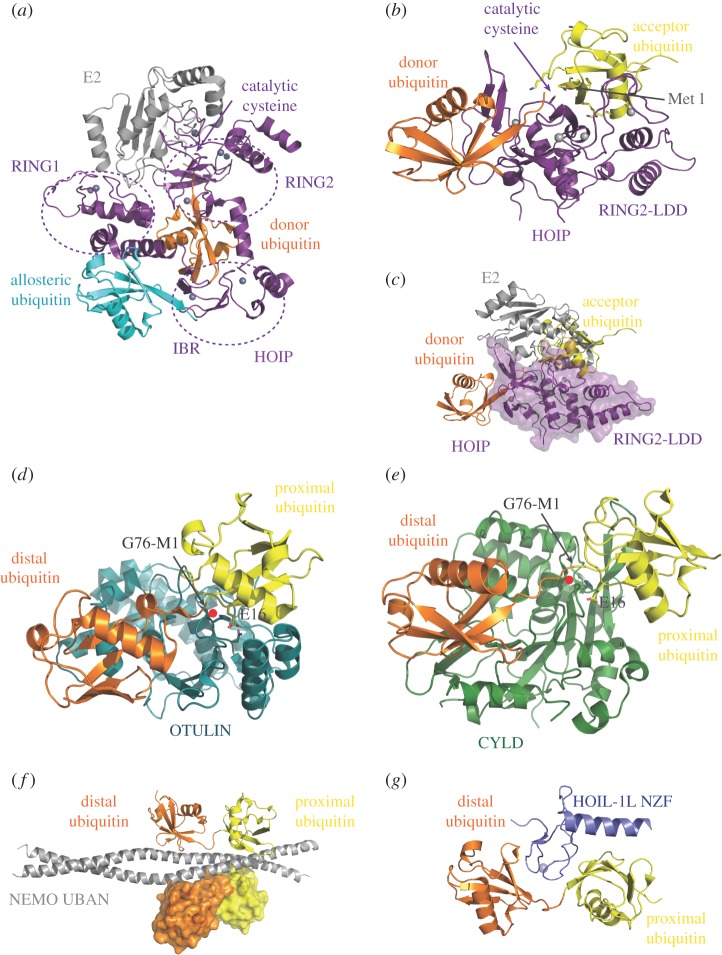
Structures of protein complexes mediating linear chain formation, disassembly and recognition. (*a*) Structure of the HOIP RBR/UBE2D2-ubiquitin complex in its monomeric form with the RBR domain shown in purple with its subdomains indicated the E2-conjugating enzyme in grey, the conjugated ubiquitin in orange and the allosteric ubiquitin in cyan (5EDV). (*b*) Structure of the active HOIP RING2-LDD/ubiquitin complex showing the position of donor (orange) and acceptor (yellow) ubiquitin. The positions of the catalytic cysteine and Met 1 of the acceptor ubiquitin are indicated (4LJO). (*c*) Clash between the binding sites of the HOIP RING2-LDD fragment for acceptor ubiquitin and E2, indicating that the E2 has to dissociate before the ubiquitin can be transferred onto the growing chain. (*d*,*e*) Structures of the OTU domain of OTULIN (*d*, 3ZNZ) and USP domain of CYLD (green) (*e*, 4WXF) bound to linear diubiquitin with the distal ubiquitin in orange and the proximal ubiquitin shown in yellow. The positions of the G76-M1 bond to be cleaved, the catalytic cysteine (red dot) and Glu 16 from the proximal ubiquitin are indicated. (*f*) Structure of the UBAN domain of NEMO (grey) bound to linear diubiquitin with the distal and proximal ubiquitin molecules in orange and yellow, respectively (2ZVO). (*g*) Structure of the NZF domain of HOIL-1L (blue) in complex with diubiquitin, showing the additional contacts made by the C-terminal helical extension (3B08).

The mechanistic features that determine the highly specific synthesis of linear ubiquitin chains have been uncovered in the high-resolution crystal structure of the RING2-LDD fragment of HOIP in complex with ubiquitin [36]. This structure contains both the donor and acceptor ubiquitins and provides a snapshot of the last step of the ubiquitination process in which ubiquitin is transferred from the E3 thioester onto the growing ubiquitin chain. The acceptor ubiquitin is accommodated by a surface created by the RING2-LDD fragment and oriented such that its N-terminal amino group is in very close proximity to the active-site cysteine (Cys 885, 3.5 Å) (figure 3*b*). In this arrangement, none of the seven lysine residues of ubiquitin is sufficiently close to Cys 885 to compete with the α-amino of Met 1 for a nucleophilic attack on the thioester intermediate, explaining the observed high linear chain specificity. Furthermore, the structure revealed that the LDD is not an isolated domain that confers specificity for linear ubiquitin chain synthesis. Instead the LDD consists of a ZF (ZF1), which is integrated into RING2 of HOIP with a helical extension (the ‘helical base’), both of which are required to correctly position the acceptor ubiquitin. This arrangement indicates that the LDD could not simply be transferred onto another E3 to bestow linear chain specificity and explains why LUBAC is the only E3 capable of forming linear ubiquitin chains. A histidine residue in the active site, close to the catalytic cysteine, has been shown to be essential for ubiquitin transfer, though not for formation of the HOIP∼ubiquitin thioester intermediate, suggesting that it acts as a general base to deprotonate the incoming nucleophile (the α-amino of Met 1) [36]. A histidine in this position is conserved in a number of RBR domains where it probably plays the same role.

The donor ubiquitin is held in place by RING2 through contacts that primarily involve hydrophobic interactions with the extended C-terminal tail of ubiquitin. Further stabilization of this arrangement is provided by a β-hairpin located within the helical base of the LDD that is specific to HOIP and together with RING2 guides the C-terminal carboxylate of Gly 76 towards the active-site cysteine of HOIP. The hydrophobic residues of RING2 contacting the donor ubiquitin are conserved in RBRs and may be a general feature of donor ubiquitin recognition by this E3 family. Remarkably, engagement of the C-terminal tail of the thioester-forming ubiquitin in an elongated conformation is also seen in HECT E3 ligases [41,42] and in E2 ∼ Ub conjugates bound to RING ligases [43–45], and may be a general mechanism to prime ubiquitin thioester intermediates for their transfer onto an amino group.

RING1 domains of RBR ligases are structurally similar to canonical RING domains and act to recognize the E2 ∼ Ub conjugate. However, recent work by Klevit and co-workers has uncovered that unlike canonical RING domains, which promote a closed conformation of the E2∼Ub conjugate, RING1 domains actively induce an open conformation of the conjugate instead [46]. Stabilization of a closed E2∼Ub conformation is an important mechanism of RING ligases to activate the conjugate for transfer of ubiquitin onto a lysine side chain [43–45]. By contrast, RBR ligases must prevent the conjugate from unproductive ubiquitin discharge onto a lysine residue to allow formation of the E3∼Ub thioester intermediate. This is achieved by inducing an open E2∼Ub conformation which allows transthiolation, an equilibrium reaction, to proceed while simultaneously suppressing reaction with an amino group [46].

Until recently, structural information on entire RBR domains was restricted to their auto-inhibited states, in which access to the E2-binding site on RING1 and/or access to the catalytic cysteine in RING2 is occluded by intramolecular interactions as manifested in HHARI and Parkin [47–50]. First insight into the architecture of an RBR domain in its active, E2∼Ub bound state was provided by the crystal structure of the RBR-LDD fragment of HOIP in complex with UBE2D∼Ub (figure 3*a*) [37]. This structure unveiled how the E2∼Ub conjugate is held in an open conformation, tightly embraced by the RBR domain, which uses multiple structural elements to contact the ubiquitin molecule and induce a conformation of the RBR in which the E2∼Ub thioester bond is brought into close proximity of the active-site cysteine in RING2. Intriguingly, the active E2∼Ub/HOIP complex in this arrangement is formed by two separate, elongated RBR molecules that contribute one RING1-IBR and RING2-LDD element each. However, this architecture was suggested to be a crystallization artefact that mimics the active state, normally formed by a monomeric RBR domain. The structure revealed some unexpected features that include the presence of an allosteric ubiquitin-binding site and extensive contacts between the E2-conjugating enzyme and RING2 that overlap with the acceptor ubiquitin-binding site. The allosteric ubiquitin is accommodated by the RING1-IBR linker and IBR domain opposite the E2-conjugated ubiquitin. Biochemical experiments indicate that binding of diubiquitin to this site enhances interaction with E2∼Ub and polyubiquitin chain synthesis, suggesting a feed-forward mechanism of linear chain formation, possibly similar to the effect of phosphorylation of ubiquitin and the Parkin UBL domain on Parkin activity [51]. On the other hand, overlapping contact sides of E2 and acceptor ubiquitin on RING2 imply that the growing polyubiquitin chain has to dissociate before another E2 ∼ Ub conjugate can be bound, thereby suppressing processive chain formation (figure 3*c*). These at-first-glance contradicting features suggest an intricate mechanism for the regulation of linear chain formation that will require further studies to be fully understood.

## 4. Disassembly of linear ubiquitin chains

All RBR ligases studied so far exist in an auto-inhibited conformation, in which catalytic activity is suppressed [34,35,47–50]. In the case of HHARI and Parkin, upstream signals are required to relieve auto-inhibition. By contrast, HOIP auto-inhibition is released upon complex formation with HOIL-1L and/or SHARPIN, and work by many groups has shown that complex formation is constitutive and that absence of either destabilizes HOIP [21–23]. This is exemplified by mutations in HOIL-1L and SHARPIN that result in loss of protein and cause disease in humans and mice, respectively (see §§6, 8 and 9 for details) [52,53]. These observations imply that LUBAC is constitutively active and that a mechanism must exist to counterbalance continuous linear chain formation, which would otherwise result in excessive signalling. Such a mechanism has been uncovered with the identification of OTULIN as a DUB specific for the hydrolysis of linear polyubiquitin chains [24,25]. OTULIN was identified independently by two different groups, in one case during the study of mutations in mice that cause embryonic angiogenic deficits [24] and in the other during a bioinformatics screen for unannotated OTU domains (figure 3*d*) [25]. OTULIN contains the catalytic triad consisting of Cys, His and Asn present in OTU members but unlike other family members is unable to hydrolyse isopeptide bonds and is instead highly specific for cleaving the peptide bond of linear polyubiquitin chains. Linkage specificity is achieved by two key features: the highly selective binding of linear diubiquitin, which is 100-fold tighter than for structurally similar Lys 63-linked diubiquitin, and from substrate-assisted catalysis, in which a residue from the proximal ubiquitin, Glu 16, contributes to the formation of the active site [25]. In the apo state of OTULIN, His 339 of the catalytic triad is contacted by a non-catalytic Asn and prevented from adopting a conformation in which it could deprotonate the catalytic cysteine. Recognition of linear diubiquitin occurs with high affinity (a *K*_D_ of 150 nM) and involves extensive contacts of OTULIN with conserved surface patches on the distal (Ile 36 and Ile 44 patches) and proximal (Phe 4 patch) ubiquitin molecules. Importantly, the proximal ubiquitin is bound in a conformation that allows its Glu 16 to be inserted into the active site to correctly position His 339 and allow linear ubiquitin chain hydrolysis to occur.

Unlike OTULIN, which is highly specific for linear ubiquitin chains, CYLD is a dual-specificity DUB that cleaves both linear and the structurally similar Lys 63-linked chains [54]. It belongs to the ubiquitin-specific protease (USP) family of DUBs but has a number of insertions and deletions, making it unique within this family. The structures of the USP of zebrafish CYLD in complex with linear (figure 3*e*) and Lys 63-linked diubiquitin show that the distal ubiquitin is bound in a similar manner in both structures that includes contacts of Leu 8 of the distal ubiquitin with a hydrophobic pocket of CYLD and extensive hydrogen bonds with the extended C-terminal tail of ubiquitin [55]. By contrast, the proximal ubiquitin moieties of linear and Lys 63 diubiquitin are recognized differently by CYLD with an approximately 13° rotation between the two forms. The β9–β10 sheet of CYLD, which is unique to CYLD, interacts with the Phe 4 centred hydrophobic patch of the proximal ubiquitin in both linkage types, while additional contacts specific to either chain type further stabilize the complex. The interaction with both the distal and proximal ubiquitin distinguishes CYLD from other, linkage-independent USP family members, and together with the flexible accommodation of the proximal ubiquitin explains how it is capable of simultaneously recognizing structurally similar linear and Lys 63 chains.

## 5. Linear ubiquitin chain recognition

Different types of polyubiquitin chains are recognized by chain linkage-specific UBDs to read the signal and translate recognition into the appropriate physiological outcome [56]. UBDs are structurally diverse and no consensus ubiquitin-binding motif exists. Similarly, UBDs contact different surfaces within ubiquitin chains to provide linkage-specific recognition. This may include recognition of surfaces on adjacent ubiquitin molecules in the chain that are only accessible in a specific linkage type, or direct recognition of the bond connecting two ubiquitin molecules. The first UBD with linear chain specificity identified was NEMO/I-κB kinase subunit gamma (IKK**γ)**, the regulatory subunit of the IKK complex [57]. The region recognizing linear ubiquitin chains was termed the ubiquitin binding in ABIN and NEMO, also called the CoZi domain (UBAN). Structural studies of the NEMO UBAN domain bound to linear diubiquitin showed that linear chain specificity relies on specific contacts between NEMO and the distal and proximal ubiquitin, yet that no direct contacts with the peptide bond connecting the two ubiquitin molecules are made (figure 3*f*) [57]. Interestingly, the specificity of the UBAN domain for linear chains (1.4 µM versus 131 µM for Lys 63 chains) [58,59] is not maintained within the full-length protein where an additional ubiquitin-binding ZF at the C-terminus of NEMO increases the affinity for Lys 63-linked chains considerably [60]. Optineurin/FIP2/NRP, another protein with a UBAN domain, is a suppressor of NF-κB activity due to its ability to compete with NEMO function [61]. It shows high sequence homology with NEMO and its UBAN domain recognizes linear ubiquitin chains in a manner that is highly similar to the interaction between NEMO and linear diubiquitin [62]. Recently, the crystal structure of another UBAN/linear ubiquitin complex has been reported, that between A20-binding inhibitor of NF-κB activation 2 (ABIN2) and linear triubiquitin [63]. While there are similarities in the mechanism of linear diubiquitin recognition to other UBAN domains, there are distinct differences. Most interestingly, the ABIN2–linear triubiquitin interaction occurs with a 2 : 1 stoichiometry in an arrangement that two ABIN2 molecules are bridged by one triubiquitin chain with the middle ubiquitin molecule in the trimer simultaneously contacting two UBAN domains. This unusual geometry suggests a model for the assembly of higher-order signalling complexes by longer polyubiquitin chains that may not only apply to ABIN2 but might be a more general model for the recognition of longer polyubiquitin chains.

Another UBD with specificity for linear ubiquitin chains is found in the DUB A20, which, in addition to the catalytic OTU domain, contains seven ZF domains. Some of these ZFs bind ubiquitin chains of varying topology, whereas others act as protein–protein interaction modules. ZF7 of A20 shows high specificity for linear chains, which is believed to protect them from removal by DUBs, which may explain its apparently opposing effect to CYLD on NF-κB activation [64,65].

Two of the LUBAC subunits, HOIL-1L and SHARPIN, contain NZF domains that recognize linear ubiquitin chains [21,22]. The interaction of the HOIL-1L NZF with linear diubiquitin differs from other NZF–ubiquitin complexes and involves a conserved sequence C-terminal to the NZF domain that adopts an α-helix and contacts the proximal ubiquitin to increase affinity of the interaction (figure 3*g*) [31]. This feature is unique to the HOIL-1L interaction. The conserved T-F/ϕ motif of the NZF contacts the hydrophobic Ile 44 patch of the distal ubiquitin, while the proximal ubiquitin uses its Phe 4 surface to contact the NZF but no contacts are made between the NZF and the linear linkage itself. Instead, linear chain specificity is achieved by contacting regions of ubiquitin that are only accessible in the linear diubiquitin conformation. At present it is not clear what the physiological relevance of the interaction of LUBAC components with linear ubiquitin chains is. It is tempting to speculate that they may play a regulatory role during chain synthesis, though no evidence for such a behaviour exists at present. Alternatively, they may help to anchor LUBAC to sites where linear chain synthesis is required and thereby help to stabilize macromolecular signalling assemblies.

## 6. Linear ubiquitin chains in the regulation of immune and cell death signalling pathways

### 6.1. Tumour necrosis factor cell signalling

**Figure 4.**
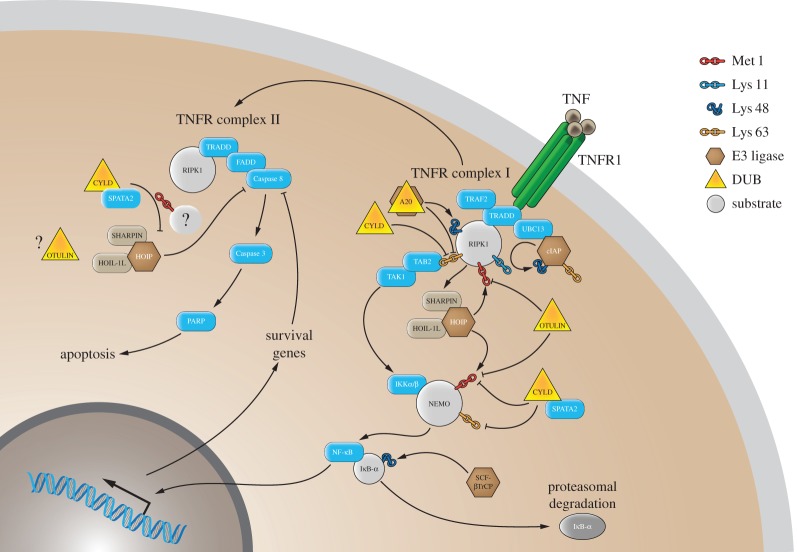
TNF-induced canonical NF-κB and apoptosis pathways. Different linkage types of ubiquitin chains, Met 1-, Lys 11-, Lys 48- and Lys 63-linked ubiquitin chains, are involved in the TNF-induced canonical NF-κB signalling pathway and the TNFR complex II-dependent apoptosis pathway. Ubiquitin chains of different linkage types are generated by E3 ligases (shown in brown), such as cIAP, HOIP-containing LUBAC complex and SCF-βTrCP. These ubiquitin chains are hydrolysed by DUBs (in yellow) such as OTULIN and CYLD. A20 has a dual role as an E3 ligase and a DUB. Ubiquitination of the substrates including cIAPs, RIPK1, NEMO and IκB-α are critical for the signalling pathway. The TNFR complex II-mediated apoptosis pathway includes RIPK1, TRADD, FADD and Caspase 8. Activation of Caspase 8 leads to the Caspase 3-dependent cleavage of PARP and apoptosis. The LUBAC complex (HOIP, SHARPIN and HOIL-1L) and the CYLD-SPATA2 complex regulates the TNFR complex II-induced apoptosis pathway. Involvement of OTULIN in this apoptosis signalling pathway is not yet determined.

One of the first signalling cascades found to be regulated by LUBAC and linear ubiquitin chains is the tumour necrosis factor (TNF)-signalling pathway (figure 4) [3,4,57]. Upon TNF stimulation, the trimeric TNF receptor (TNFR) recruits a number of signalling molecules including TNFRSF1A associated via death domain (TRADD), TNF receptor associated factor 2 (TRAF2), receptor-interacting protein kinase 1 (RIPK1) and cellular inhibitor of apoptosis protein 1/2 (cIAP1/2). The recruitment of LUBAC components, HOIP, HOIL-1L and SHARPIN to the TNFR complex I has been shown by Walczak and co-workers using moTAP-TNF pull-down assays followed by mass spectrometry analysis [4,22]. In this signalling pathway, LUBAC linearly ubiquitinates its substrates, NEMO and RIPK1 [3,22]. Once linear ubiquitin chains are formed by LUBAC, the NEMO-UBAN domain (see §5) interacts with the chains and subsequently activates NF-κB [57]. In addition to NF-κB activation, LUBAC components HOIL-1L and SHARPIN regulate the TNF-induced JUN N-terminal kinase (JNK) pathway. It was shown that TNF-induced JNK activation is suppressed in HOIL-1L knockdown MCF-7 cells and SHARPIN-deficient chronic proliferative dermatitis mutation (Cpdm) mouse embryonic fibroblasts (MEFs) [4,21,22]. Thus far, substrates of LUBAC regulating JNK are unknown and the detailed mechanisms by which JNK activation is mediated by linear ubiquitination require further studies.

In the TNF signalling cascade, deubiquitinases such as CYLD, OTULIN and A20 [25,29,30,32,33,64,65] negatively regulate NF-κB activation. As described in §4, both OTULIN and CYLD form a complex with the HOIP-PUB domain and this interaction is important for the negative regulation of the NF-κB signalling pathway [26]. When a HOIP-PUB mutant, which is not able to interact with these DUBs, is reconstituted in HOIP-null cells, TNF-induced NF-κB activation is up-regulated [32], suggesting that both OTULIN and CYLD are counterbalancing LUBAC-dependent activation of downstream signalling by forming a complex with HOIP. Importantly, the HOIP-SPATA2-CYLD complex is formed at the TNFR complex, whereas OTULIN does not translocate with LUBAC to the TNFR complex [26,29,33]. As both CYLD and OTULIN negatively regulate the NF-κB signalling cascade, the impact of these deubiquitinases in different LUBAC-dependent signalling cascades may be context-dependent. More recent studies have shown the importance of OTULIN in mice. Immune cell-specific depletion of OTULIN leads to cell-type specific effects: constitutive active NF-κB signalling and overproduction of cytokines in myeloid cells, and down-regulation of linear ubiquitination signal in the B and T cells based on LUBAC degradation [66]. OTULIN and CYLD were also shown to be important regulators of the muramyl dipeptide (MDP)-induced innate immune signalling cascade mediated through RIPK2-ubiquitination [27]. Among the DUBs regulating the NF-κB signalling pathway, A20, which is also known to have E3 ligase activity, has a linear ubiquitin chain interaction domain outside of its catalytic region (as described in §5). The A20-ZF7 interaction with linear Ub chains is crucial for the recruitment of A20 in the TNFR complex, and regulation of TNF-dependent NF-κB activation [64,65]. However, A20 regulates this signalling pathway through its interaction with linear ubiquitin chains and not via its catalytic activity, which distinguishes it from CYLD and OTULIN and implies that its effect is dependent on LUBAC-generated linear ubiquitin chains.

Collectively, LUBAC components, DUBs including OTULIN, CYLD and A20, and linear ubiquitination are critical in many immune response pathways.

### 6.2. Cell death signalling

Other cell signalling cascades that are regulated by LUBAC are cell death pathways [7,10,67,68]. SHARPIN-deficient Cpdm MEFs are significantly susceptible to TNF-dependent induction of apoptosis (figure 4) [21,22], and TNF-treated HOIL-1L-deficient mice revealed that apoptosis in liver is up-regulated [3]. Furthermore, recent studies both *in vivo* and *in vitro* showed that HOIP also plays a critical role in the regulation of apoptosis [69–74]. While substrates for linear ubiquitination during apoptosis signalling remain unclear, it has been established that each of the LUBAC components and LUBAC catalytic activity is required for its anti-apoptosis function [69]. More recent work revealed that HOIP is cleaved by effector Caspases (Caspase 3 and Caspase 6) upon TNF stimulation [75]. Cleavage sites predicted are Asp 348, Asp 387 and Asp 390, resulting in an N-terminal fragment containing PUB-ZF-NZF1 and a C-terminal fragment containing NZF2-UBA-RBR-LDD. The authors demonstrated that cells expressing cleavage-resistant HOIP mutant are protected from TNF combined with cycloheximide (CHX)-induced apoptosis. The remaining question is whether TNF-induced apoptosis by HOIP cleavage involves a linear ubiquitination event. The SPATA2-CYLD complex also plays a role in the TNF-dependent apoptosis pathway [76]. Different studies highlighted that TNF-induced apoptosis due to LUBAC deficiency is dependent on the TNFR complex II pathway (figure 4) [10,67,68]. For example, it was shown that targeting TNFR complex II components by the expression of dominant negative FADD or Caspase 8 inhibitor, CrmA in Cpdm MEFs inhibits apoptosis, suggesting that apoptosis is mediated through the TNFR complex II [21].

Cpdm mice, which have a nonsense mutation in the *SHARPIN* gene, suffer from systemic inflammation [53]. In these mice, secondary lymphoid organ development and differentiation of T cells and B cells are defective, leading to deficiency of IgG production [53]. Especially the skin inflammation phenotype accompanied with apoptosis observed in Cpdm mice is largely rescued by epithelial-specific TRADD deficiency, and epithelial-specific Fas-associated protein with death domain (FADD) and RIPK3 deficiency [70,71]. These observations suggest that apoptosis plays a major role in the regulation of skin inflammation caused by SHARPIN deficiency. On the other hand, because deficiency of the necroptosis regulators RIPK3 and mixed lineage kinase domain-like protein (MLKL) had only mild effects on skin inflammation, necroptosis has a minor role in this model. The skin inflammatory phenotype and induction of cell death in skin tissue observed in Cpdm mice was largely rescued by double genetic ablation of Caspase 1 and Caspase 11, whereas inflammation in other organs remained [77]. An independent study revealed functional differences between Caspase 1 and Caspase 11 by using Caspase 1-deficient (*Caspase 1^−/−^*; Caspase 11 transgenic) mice, showing that Caspase 1 has a major role in the regulation of inflammatory phenotype in Cpdm mice [78]. Mechanistically, SHARPIN directly interacts with Caspase 1 and negatively regulates the activity of Caspase 1, leading to the suppression of mature IL-1β and IL-18 production. Whether the role of SHARPIN in Caspase 1/11-dependent skin inflammation depends on the catalytic function of LUBAC remains to be understood. The cross of HOIL-1-L-deficient mice with Cpdm mice leads to embryonic lethality at the embryonic stage E10.5 accompanied with up-regulation of apoptosis, identical to *HOIP^Δlinear/Δlinear^* mice in which the catalytic RBR region is deleted [79]. These observations suggest cooperative roles of HOIL-1-L and SHARPIN in mouse embryonic development and apoptosis. Similar to *HOIP^Δlinear/Δlinear^* mice, HOIP-deficient mice and HOIP catalytic inactive mutant knockin mice are embryonic lethal at E10.5 [40,69], indicating a critical role of HOIP catalytic activity in regulation of mouse embryonic development and apoptosis.

The role of LUBAC in the cell death signalling cascade is not limited to the TNF-induced pathway; Walczak and co-workers have shown both *in vitro* and *in vivo* that polyI:C-induced Toll-like receptor 3 (TLR3)-dependent apoptosis also involves LUBAC [80]. It was also demonstrated that HOIP plays an anti-apoptosis role in the cisplatin-induced genotoxicity pathway via the protein kinase ataxia–telangiectasia mutated (ATM) [72].

These observations collectively indicate a critical role of LUBAC and linear ubiquitination in the regulation of various apoptosis signalling pathways.

### 6.3. Other immune signalling pathways

It is well established by now that LUBAC plays an important role in multiple types of immune responses, including activation of NF-κB induced by various ligands, such as peptidoglycan (PGN), CD40 ligand (CD40-L), interleukin 1beta (IL-1β) and lipopolysaccharide (LPS) in B cells or in macrophages [21,22,81,82]. For example, PGN-dependent induction of the nucleotide-binding oligomerization domain-containing protein 1/2 (NOD1/2) pattern recognition receptor signalling cascade leads to linear ubiquitination of RIPK2 by recruiting LUBAC into the receptor complex [81,82]. In this signalling cascade, LUBAC ligase activity is required for efficient NF-κB activation and secretion of pro-inflammatory cytokines. As previously described (in §3), Lys 63–Met 1 hybrid ubiquitin polymers are formed on the substrates, such as NEMO in the IL-1β immune signalling cascades, RIPK2 via the NOD2 pathway and RIPK1 via TLR3 or TNFR [40,83]. These are very interesting observations suggesting that LUBAC-induced linear ubiquitination may depend on the initial Lys 63 ubiquitin chain formation on the substrates.

LUBAC is also involved in the regulation of TLR signalling cascades. One of the first studies that linked TLR signalling and SHARPIN was based on a system-level analysis of TLR-stimulated macrophages [84]. The authors demonstrated that macrophages derived from Cpdm mice were defective in IL-12 production induced by TLR activation, and that the effects of SHARPIN deficiency on the TLR2-induced transcriptome were highly correlated with the effects of the hypomorphic Leu 153 Pro/panr2 point mutation in the gene encoding NEMO. Furthermore, HOIP was demonstrated to be involved in the TLR3-induced signalling cascade examined in HOIP-deficient HaCaT or MEFs [80,83], as well as HOIL-1L in the TLR4 signalling pathway using an LPS-induced systemic inflammation model in HOIL-1L-deficient mice [85] and in a mouse B-cell line [86].

Additional roles for LUBAC during innate immune signalling have been described including NEMO ubiquitination in the MAVS-TRAF3 complex [87], IRF3 ubiquitination in the RLR-induced IRF-3-mediated pathway of apoptosis (RIPA) [88] and ASC ubiquitination in the NLRP3/ASC inflammasome signalling pathway [85]. Furthermore, it has been suggested that LUBAC plays a role in LMP1 signalling [89], and in the RIG-I TRIM25-mediated IFN pathway [90]; however, it is not yet clear at present if this requires LUBAC catalytic activity, and the mechanistic understanding of LUBAC function in these pathways awaits further studies. Recent work from multiple groups showed an important function of mucosa associated lymphoid tissue lymphoma translocation gene 1 (MALT1)-dependent HOIL-1L cleavage in the negative regulation of the T-cell receptor (TCR)-induced NF-κB signalling cascade [91–94]. The MALT-1-cleaved products of HOIL-1L are an N-terminal fragment containing the UBL, and a C-terminal fragment containing the NZF and RBR domains. Although both of these HOIL-1L protein fragments are stable in cells, cleavage leads to destabilization of HOIP [91]. This is rather surprising because the HOIL-1L UBL domain is sufficient to interact with HOIP. Based on these studies, we speculate that there might be an additional interplay between HOIP and HOIL-1L that is not understood yet. In the TCR signalling pathway, it was also shown that the B-cell lymphoma/leukaemia 10 (Bcl10) is linearly ubiquitinated and recognized by NEMO. Caspase recruitment domain family member 11 (CARD11) and MALT1 are both required for the TCR-induced linear ubiquitination of Bcl10 as demonstrated in CARD11- and MALT1-deficient Jurkat T cells [95]. To fully understand the functional relationship between MALT1-dependent HOIL-1-L cleavage and subsequent inactivation of NF-κB signalling and Bcl10 ubiquitination, further studies are required.

LUBAC plays a critical role in late thymocyte differentiation, FOXP3+ regulatory T (Treg)-cell development and Treg cell homeostasis [96]. Treg-specific HOIP ablation leads to lethal immune pathology in mice [96]. Furthermore, the HOIP-RBR region regulates T-cell and B-cell differentiation. In T-cell-specific HOIP^Δlinear^ mice, in which HOIP-RBR is deleted, thymic CD4^+^ or CD8^+^ T-cell numbers were markedly reduced with severe defects in NKT cell development [73]. In B cells derived from B-cell-specific HOIP^Δlinear^ mice, the development of the B1 cells is defective and CD40- or TACI-induced canonical NF-κB and ERK signalling pathways are impaired [97]. Collectively, these studies show that LUBAC plays an important role in adaptive immunity.

## 7. Linear ubiquitination in the non-immune signalling cascades

In addition to its well-established role in the regulation of immune signalling and apoptosis (as described in §6), it is becoming apparent that linear ubiquitin chains also have other cellular roles.

### 7.1. Wnt signalling pathway regulated by OTULIN

Linear ubiquitination regulators are involved in non-immune signalling cascades as illustrated in Gumby mice, in which the *OTULIN* gene has missense mutations that are embryonic lethal due to vascular formation defects [24]. The interaction of OTULIN with a key component Dishevelled (Dvl/Dsh) mediates this regulation through the Wnt signalling cascade.

### 7.2. Genotoxic stress-induced NF-κB activation by LUBAC

The LUBAC components were shown to be important for NF-κB activation induced by genotoxic stress [98]. Genotoxic stress in cells leads to linear ubiquitination of NEMO in the cytosolic fraction, which requires LUBAC, and subsequent activation of NF-κB. The molecular mechanism that mediates LUBAC activation upon genotoxic stress remains to be understood.

### 7.3. Linear ubiquitination by *Drosophila* linear ubiquitin E3 ligase in heat tolerance

**Figure 5.**
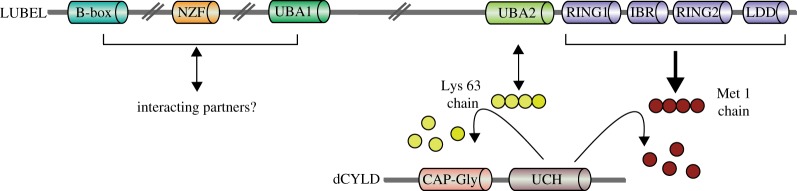
Domain structures of *Drosophila* LUBEL and dCYLD. *Drosophila* LUBEL-RBR-LDD is the catalytic region for linear ubiquitination. The UBA2 domain interacts with Lys 63-linked ubiquitin chains. An N-terminal region of LUBEL may interact with unknown interacting partners. UBA2 interacts with Lys 63-linked ubiquitin chains and dCYLD cleaves linear and Lys 63-linked ubiquitin chains. Npl4 zinc finger (NZF), ubiquitin (Ub)-associated (UBA1 and UBA2), RING between RING (RBR)-C. The RBR-C region consists of RING1, in-between RING (IBR), RING2 and linear Ub chain-determining domain (LDD), cytoskeletal-associated protein-glycine-conserved (CAP-Gly) domain and ubiquitin carboxyl-terminal hydrolase (UCH). The double bars between domains indicate that they are separated by large stretches of sequence, resulting in LUBEL being almost three times larger (2892 amino acids) than human HOIP (1072 amino acids).

More recently, a ubiquitin E3 ligase in *Drosophila*, which specifically generates linear ubiquitin chains, was identified [99]. *Drosophila* linear ubiquitin E3 ligase (LUBEL)/CG11321 consists of a highly conserved RING1-RBR-RING2-LDD catalytic domain, and protein–protein interaction domains including a B-box, NZF and two UBAs (figure 5). In comparison with human HOIP, the N-terminal region of LUBEL is stretched out with large insertions between recognizable domains, and LUBEL is approximately three times larger. Furthermore, some of the protein–protein interaction domains present in human HOIP (PUB, ZF and one of the NZF domains) are not present in LUBEL. Based on bioinformatic analysis, genes for the LUBAC subunits, namely SHARPIN or HOIL-1L, and the linear ubiquitin-specific DUB, OTULIN, are not predicted in the *Drosophila* genome. Even without these subunits, LUBEL alone generates linear ubiquitin chains in a RING/HECT hybrid manner. The DUB for linear chains in *Drosophila* is dCYLD, which was confirmed to cleave linear and Lys 63-linked ubiquitin chains, similar to human CYLD. In LUBEL, the PUB domain, which is responsible for recruiting CYLD and SPATA2 in human HOIP, is lacking. Instead, the LUBEL C-terminal region (the RBR-LDD domain) interacts with recombinant dCYLD *in vitro*. An orthologue of SPATA2 in *Drosophila* called Tamo was shown to have a conserved PUB domain [100] and it is of high interest to understand if Tamo is involved in the regulation of linear ubiquitination in *Drosophila*. These observations suggest that, in *Drosophila*, LUBEL and dCYLD play an important role in the regulation of linear ubiquitin chain formation and destruction. In contrast with HOIP knockout or catalytic inactive knockin mice that are embryonic lethal, catalytic inactive knockin LUBEL flies are viable and showed no obvious developmental defect. LUBEL knockdown flies and LUBEL catalytic inactive mutant flies, in which linear ubiquitination is below the detection limit, are defective in heat tolerance, which may be relevant also in the mammalian system.

These observations suggest that there is more to learn about the roles of LUBAC and linear ubiquitination in the mammalian system in addition to their roles in immune and apoptotic signalling.

## 8. The role of the LUBAC component SHARPIN independent of catalytic activity

The LUBAC subunit, SHARPIN was shown to interact with integrin and inhibit β-integrin activity [101]. This interaction is mediated by the SHARPIN UBL domain and important residues for the interaction with integrin are shared with the HOIP interaction [102]. Because LUBAC forms a relatively stable complex and depletion of a LUBAC component in cells often leads to destabilization of the other complex components, it will be interesting to understand how the integrin–SHARPIN complex is formed, and how SHARPIN may determine its interacting partner in cells, namely HOIP or integrin. Recently, it was demonstrated that SHARPIN controls mouse mammary gland development [103]. Whether these events depend on the ubiquitination function of the LUBAC complex or alternatively depend on the role of SHARPIN alone remains to be understood.

## 9. Linear ubiquitin chains and disease

### 9.1. Human diseases associated with mutations in HOIP and HOIL-1L

In a patient with multi-organ auto-inflammation, combined immunodeficiency, subclinical amylopectinosis and systemic lymphangiectasia, the *HOIP* gene is homozygous for a mutation at Leu 72 Pro (L72P) in the PUB domain (described in §2) [104] (table 1). The HOIP L72P mutation leads to destabilization of HOIP itself and LUBAC. Both IL-1β and TNF-induced NF-κB activation and linear ubiquitination are impaired in fibroblasts derived from the patient. However, the patient's monocytes respond to IL-1β more vigorously than control monocytes, whereas the activation and differentiation of the patient's B cells are impaired in response to CD40 engagement. These cellular and clinical phenotypes largely overlap those of HOIL-1L-deficient patients. For example, it was reported that a new fatal human inherited disorder characterized by chronic auto-inflammation, invasive bacterial infections and muscular amylopectinosis carried biallelic loss-of-expression and loss-of-function mutations in HOIL-1L (RBCK1) [52]. Moreover, *RBCK1* gene mutations were identified in non-immune disease patients, namely in glycogen storage disease patients, who suffer from myopathy and cardiomyopathy. It was found that 10 patients from eight different families had heterozygous missense or truncating mutations in the *RBCK1* gene [105,106]. These observations suggest a role of HOIL-1L in the regulation of non-immune pathology and cellular functions, which are independent of immune system-dependent NF-κB activation.

HOIP has also been shown to be involved in activated B-cell-diffuse large B-cell lymphoma (ABC-DLBCL). Two germ-line polymorphisms affecting HOIP are rare among healthy individuals but enriched in ABC-DLBCL. These are located in the UBA of HOIP that mediates the interaction with HOIL-1-L (see §2), and have been suggested to increase their association, thereby leading to up-regulation of NF-κB [109]. Activation of IKK-dependent NF-κB through the CARD11-MALT1-BCL10 (CBM) complex in the chronic active B-cell receptor (BCR) signalling cascade, which is a hallmark of ABC-DLBCL, includes the ubiquitin E3 ligases cIAP1, cIAP2 as well as LUBAC. cIAP1/2-induced Lys 63 ubiquitin chains on cIAPs and Bcl10 recruits LUBAC to the signalling complex, which is crucial for subsequent IKK activation [108]. This aligns with the previous finding in the TNFR signalling cascade that the E3 ligase activity of cIAPs is required for LUBAC recruitment into the receptor complex.

### 9.2. OTULIN-related auto-inflammatory syndrome

Two independent groups reported that homozygous gene mutations of OTULIN/FAM105B were found in autoimmune disease patients [66,107] (table 1). Loss of function mutations or a missense mutation in human *OTULIN* causes a potentially fatal auto-inflammatory condition, which was termed OTULIN-related auto-inflammatory syndrome (ORAS). Expression of immunologically related genes in whole blood and fibroblasts derived from patients was high in comparison to the cells from a healthy patient. The mutations found in these patients, Tyr 244 Cys and Leu 272 Pro, are located near or on the S1 distal ubiquitin binding site [107]. It was confirmed that the Leu 272 Pro mutation abolishes the catalytic activity of OTULIN [66].

**Table 1.** Reported mutations in *RNF31*, *RBCK1* and *FAM105B* genes in human patients.

gene name	protein name	gene mutations (amino acid alterations)	protein domains affected	symptoms	reference
*RNF31*	HOIP	L72P (homozygous)	PUB	multi-organ auto-inflammation, combined immunodeficiency, subclinical amylopectinosis, and systemic lymphangiectasia	[104]
*RBCK1*	HOIL-1L	L41fsX7 (homozygous)	all domains (a mutation at the N-terminus to UBL)	chronic auto-inflammation, invasive bacterial infections and muscular amylopectinosis	[52]
		Q185X;c.ex1_ex4del (compound heterozygous)	NZF-RING1-IBR-RING2 (a mutation at the N-terminus to NZF); UBL (deletion of 1-154)	chronic auto-inflammation, invasive bacterial infections and muscular amylopectinosis	[52]
		E243X; N387S (compound heterozygous)	RING1-IBR-RING2 (a mutation between NZF and RING1); IBR	myopathy and cardiomyopathy	[105]
		E299VfsX18 (homozygous)	RING1-IBR-RING2 (a mutation within RING1)	myopathy and cardiomyopathy	[105]
		A241GfsX34 (homozygous)	RING1-IBR-RING2 (a mutation between NZF and RING1)	myopathy and cardiomyopathy	[105]
		A18P (homozygous)	N-terminus	myopathy and cardiomyopathy	[105]
		E243GfsX114; c.ex1_ex4del (compound heterozygous)	RING1-IBR-RING2(a mutation between NZF and RING1); UBL (deletion of 1-154)	myopathy and cardiomyopathy	[105]
		R352X (homozygous)	IBR	myopathy and cardiomyopathy	[105]
		R298RfsX40 (homozygous)	RING1	myopathy and cardiomyopathy	[105]
		R165RfsX111 (homozygous)	NZF-RING1-IBR-RING2 (a mutation between UBL and NZF)	myopathy and cardiomyopathy	[105]
		Q222X;E190fs (compound heterozygous)	NZF-tail-RING1-IBR-RING2 (a mutation between NZF and RING1); NZF-RING1-IBR-RING2	progressive muscular weakness and cardiomyopathy	[106]
*FAM105B*	OTULIN	L272P (homozygous)	OTU	auto-inflammatory syndrome	[66,107]
		Y244C (homozygous)	OTU	auto-inflammation, paniculitis and dermatosis	[107]
		G174DfsX2 (homozygous)	OTU	auto-inflammation, paniculitis and dermatosis	[107]

### 9.3. Lupus/autoimmune diseases, the E2 enzyme UBE2L3 and linear ubiquitin chains

There are several reports linking a single risk haplotype across the *UBE2L3/UBCH7* gene with systemic lupus erythematosus and autoimmune diseases [110,111]. UBE2L3/UBCH7 specifically acts with HECT-type and RBR family E3 ubiquitin ligases including HOIP and HOIL-1L. Based on a single risk haplotype across UBE2L3, it was hypothesized that UBE2L3 together with LUBAC regulates NF-κB. Indeed, it was shown that UBE2L3 is the preferred E2-conjugating enzyme for LUBAC *in vivo*, and that UBE2L3 is essential for LUBAC-mediated activation of NF-κB. How LUBAC recognizes UBE2L3 over other E2s such as UBE2D is yet to be clarified.

## 10. Conclusion

Since the first biochemical study of LUBAC-induced linear ubiquitination was reported by Iwai and co-workers in 2006 [2], our understanding of LUBAC and linear ubiquitination has expanded significantly from molecular and cellular functions to its impact on *in vivo* roles and human diseases. Linear ubiquitin chains belong to those polyubiquitin chain types that are not primarily used for proteasomal degradation of proteins but rather used as signalling tags to increase the functional variety in regulatory mechanisms of cellular responses. Understanding the molecular specificity of linear ubiquitination, namely (i) how and which enzymes generate or disassemble the chains, (ii) how substrates are chosen for linear ubiquitination, and (iii) how linear ubiquitin chains are recognized, will allow a better dissection of the biology regulated by linear ubiquitination. Great progress has been made in recent years with the discovery of novel regulators of LUBAC, the finding that ubiquitin chains can be formed as hybrids of linear and Lys 63 chains and the association of mutations in LUBAC with human disease, and it will be exciting to see where the linear ubiquitin chain will take us next.
